# Dynamic Contrast-Enhanced Magnetic Resonance Imaging for the Prediction of Monoclonal Antibody Tumor Disposition

**DOI:** 10.3390/ijms23020679

**Published:** 2022-01-08

**Authors:** Brandon M. Bordeau, Joseph Ryan Polli, Ferdinand Schweser, Hans Peter Grimm, Wolfgang F. Richter, Joseph P. Balthasar

**Affiliations:** 1 Department of Pharmaceutical Sciences, University at Buffalo, 450 Pharmacy Building, Buffalo, NY 14214, USA; bmbordea@buffalo.edu (B.M.B.); jrpolli@buffalo.edu (J.R.P.); 2 Buffalo Neuroimaging Analysis Center, Department of Neurology, School of Medicine and Biomedical Sciences, University at Buffalo, Buffalo, NY 14203, USA; schweser@buffalo.edu; 3 Clinical and Translational Science Institute, Center for Biomedical Imaging, University at Buffalo, Buffalo, NY 14203, USA; 4 Roche Pharmaceutical Research and Early Development, Pharmaceutical Sciences, Roche Innovation Center Basel, F. Hoffmann-La Roche Ltd., Grenzacherstrasse 124, 4070 Basel, Switzerland; hans_peter.grimm@roche.com (H.P.G.); wolfgang.richter@roche.com (W.F.R.)

**Keywords:** dynamic contrast enhanced-magnetic resonance imaging, physiologically based pharmacokinetic modeling, monoclonal antibody, tumor pharmacokinetics

## Abstract

The prediction of monoclonal antibody (mAb) disposition within solid tumors for individual patients is difficult due to inter-patient variability in tumor physiology. Improved a priori prediction of mAb pharmacokinetics in tumors may facilitate the development of patient-specific dosing protocols and facilitate improved selection of patients for treatment with anti-cancer mAb. Here, we report the use of dynamic contrast-enhanced magnetic resonance imaging (DCE-MRI), with tumor penetration of the contrast agent gadobutrol used as a surrogate, to improve physiologically based pharmacokinetic model (PBPK) predictions of cetuximab pharmacokinetics in epidermal growth factor receptor (EGFR) positive xenografts. In the initial investigations, mice bearing Panc-1, NCI-N87, and LS174T xenografts underwent DCE-MRI imaging with the contrast agent gadobutrol, followed by intravenous dosing of an ^125^Iodine-labeled, non-binding mAb (8C2). Tumor concentrations of 8C2 were determined following the euthanasia of mice (3 h–6 days after 8C2 dosing). Potential predictor relationships between DCE-MRI kinetic parameters and 8C2 PBPK parameters were evaluated through covariate modeling. The addition of the DCE-MRI parameter K^trans^ alone or K^trans^ in combination with the DCE-MRI parameter Vp on the PBPK parameters for tumor blood flow (QTU) and tumor vasculature permeability (σ_TU_^V^) led to the most significant improvement in the characterization of 8C2 pharmacokinetics in individual tumors. To test the utility of the DCE-MRI covariates on a priori prediction of the disposition of mAb with high-affinity tumor binding, a second group of tumor-bearing mice underwent DCE-MRI imaging with gadobutrol, followed by the administration of ^125^Iodine-labeled cetuximab (a high-affinity anti-EGFR mAb). The MRI-PBPK covariate relationships, which were established with the untargeted antibody 8C2, were implemented into the PBPK model with considerations for EGFR expression and cetuximab-EGFR interaction to predict the disposition of cetuximab in individual tumors (a priori). The incorporation of the K^trans^ MRI parameter as a covariate on the PBPK parameters QTU and σ_TU_^V^ decreased the PBPK model prediction error for cetuximab tumor pharmacokinetics from 223.71 to 65.02%. DCE-MRI may be a useful clinical tool in improving the prediction of antibody pharmacokinetics in solid tumors. Further studies are warranted to evaluate the utility of the DCE-MRI approach to additional mAbs and additional drug modalities.

## 1. Introduction

Personalized medicine aims to improve patient outcomes through the selection of therapies and doses that are rationally defined based on patient-specific characteristics. For cancer therapy, monoclonal antibodies (mAbs) are used to specifically target tumor-associated antigens, and patients eligible for mAb therapy are often identified through tumor antigen profiling [1]. Although more than 20 mAbs have been approved for solid tumor indications, and although there are 44 anti-cancer mAbs undergoing late-stage clinical development [2], there has been little success in the development of methods capable of meaningful a priori prediction of mAb tumor pharmacokinetics in individual patients. Mechanistic mathematical models, including physiologically based pharmacokinetic (PBPK) models, have shown some promise in predicting mean mAb pharmacokinetics in preclinical animal models and in humans [3,4,5,6]; however, 90% confidence intervals for predicted concentrations often span several orders of magnitude owing to the unexplained inter-subject variability in the determinants of mAb tumor disposition. As such, present models hold little value in predicting the anti-tumor efficacy of mAb in individual patients [4,7]. The variability in mAb tumor pharmacokinetics may relate to inter-patient and/or inter-tumor variability in tumor antigen expression and turnover, tumor blood flow, the porosity of tumor vessels, hydrostatic and oncotic pressure gradients, and variability in the composition of tumor stroma [8,9,10].

During the course of the clinical development of drugs, including mAb, effort is often put in to improve patient-specific predictions of pharmacokinetics and pharmacodynamics (PK/PD) through the use of “population” PK/PD modeling, where variability in model parameters is explained, in part, through consideration of variability in patient characteristics that are known or readily available (age, weight, creatinine clearance, etc.). Relationships between model parameters and patient characteristics (termed “covariates”) are defined and then subsequently employed to improve a priori predictions of drug PK/PD and to assist in the selection of optimal dosing regimens for individual patients [11,12,13]. Covariates that can improve the a priori prediction of mAb disposition in solid tumors are generally unknown or are not readily available. Some patient-specific information can be gathered through post-biopsy assays, such as tumor antigen expression; however, prior PK model sensitivity analysis has demonstrated that mAb tumor disposition is highly dependent on parameters relating to passive transport processes, such as vascular permeability [14,15], which cannot be assessed with post-biopsy assays.

**Figure 1:**
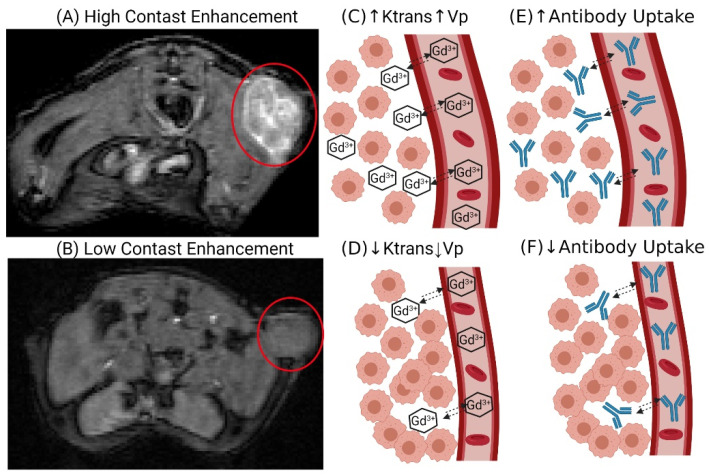
DCE-MRI: The DCE-MR images on the left side provide examples of a tumor with high contrast enhancement (**A**) and low contrast enhancement (**B**) following gadobutrol injection. High contrast enhancement is the result of greater gadobutrol uptake into the tumor (**C**), which results in larger values for Ktrans and Vp relative to the low contrast-enhancing tumor (**D**). The DCE-MRI parameters are representative of tumor physiology (vascular permeability, plasma volume, and blood flow) that is known to impact mAb disposition; therefore, the high contrast-enhancing tumor is expected to have greater mAb uptake (**E**) relative to the low contrast-enhancing tumor (**F**). Created with BioRender.com (accessed on 23 Novermber 2021).

**Figure 2:**
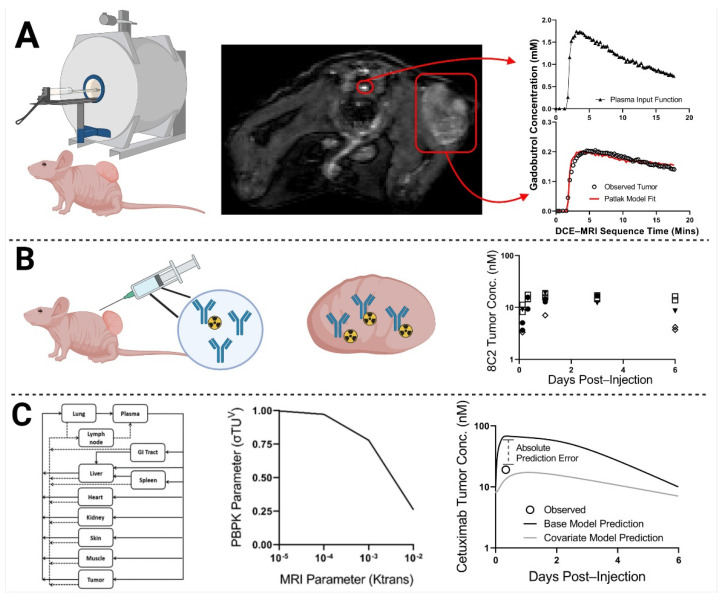
Workflow Diagram: In step (**A**), xenograft-bearing mice underwent DCE-MRI. Images were analyzed to obtain Patlak pharmacokinetic model fits for each xenograft bearing mouse, using individual plasma input functions. In step (**B**), one day post-MRI, the same xenograft-bearing mice were administered the non-tumor binding antibody 8C2 with a tracer dose of ^125^I-8C2 (depicted as a blue Y with a radioactive symbol). Following tumor resection, 8C2 tumor concentrations were determined via gamma counting at a single terminal timepoint per mouse (timepoints ranged from 3 h to 6 days). In step (**C**), relationships were drawn through PBPK modeling between the observed quantitative MRI parameter values for each tumor (obtained in step (**A**)) and the corresponding observed value for 8C2 tumor concentration (obtained in step (**B**)). The middle image for step C represents a potential relationship between the MRI parameter K^trans^ and the PBPK parameter that represents tumor vascular permeability (σTU^V^). A PBPK model that implemented the MRI-PBPK relationship shown in step (**C**) would predict less tumor uptake (due to a higher reflection coefficient) for a xenograft with an observed K^trans^ value of 10^−5^ min^−1^ in comparison to a tumor with a higher observed K^trans^ value of 10^−2^ min^−1^. A second round of MRI imaging is then performed for xenograft-bearing mice that are administered cetuximab (repeating steps (**A**,**B**)). Cetuximab tumor uptake is predicted a priori for individual xenografts based on their observed DCE-MRI parameter values using the MRI-PBPK relationships that were established in step (**C**). Model prediction accuracy for the MRI-PBPK covariate model was compared to the predictions that were made using the base PBPK model without consideration of the MRI-PBPK relationship. Created with BioRender.com (accessed on 23 Novermber 2021).

The objective of the presented work was to determine whether the kinetics of movement of contrast agents into and within tumors, as assessed by dynamic contrast-enhanced magnetic resonance imaging (DCE-MRI), may be used as a covariate to improve the prediction of mAb uptake and disposition within solid tumors in individual subjects. DCE-MRI has been widely employed in the clinic to detect tumor lesions and, in some cases, to monitor response to anti-cancer therapy [16,17]. DCE-MRI assesses the time course of a T1-shortening contrast agent through a series of T1-weighted scans. Kinetic models are applied to the DCE-MRI scans to determine tumor-specific parameters describing the disposition of the contrast agent. Standard DCE-MRI parameters include the volume transfer constant (K^trans^), reflux rate constant (Kep), and the plasma volume ratio (Vp or Fpv) [18]. The K^trans^ parameter represents the transport of the contrast agent from the vascular space and is dependent on the tumor vessel permeability, vascular surface area, and blood flow [18,19]. Kep is the ratio of K^trans^ and the extracellular volume ratio (Ve) and is a measure of the tumor interstitial volume [18,19]. Many pre-clinical investigations have developed direct relationships between tumor physiologies that are known to impact mAb disposition and the quantitative parameters obtained from DCE-MRI analyses. For example, V_e_ was correlated to the collagen content of xenograft models of pancreatic cancer [20]. Tumor collagen content has been inversely related to the uptake and penetration of mAb in solid tumors [21]. Additionally, the kinetic parameter K^trans^ has been related to tumor interstitial fluid pressure [22] and employed as a predictor of anti-tumor response to anti-angiogenic therapies [23,24,25]. Thus, prior work has demonstrated that DCE-MRI analyses may be used as a probe of tumor characteristics that are known determinants of mAb uptake and disposition, suggesting that MRI parameters obtained from DCE-MRI may have utility as covariates for improving mathematical model predictions of antibody pharmacokinetics in solid tumors a priori. Figure 1 provides a graphical representation of the relationship between DCE-MRI and mAb tumor uptake, and Figure 2 provides a graphical schematic of the steps used in this work.

## 2. Results

### 2.1. Antibody Tumor Pharmacokinetics and a Priori PBPK Model Predictions

**Figure 3:**
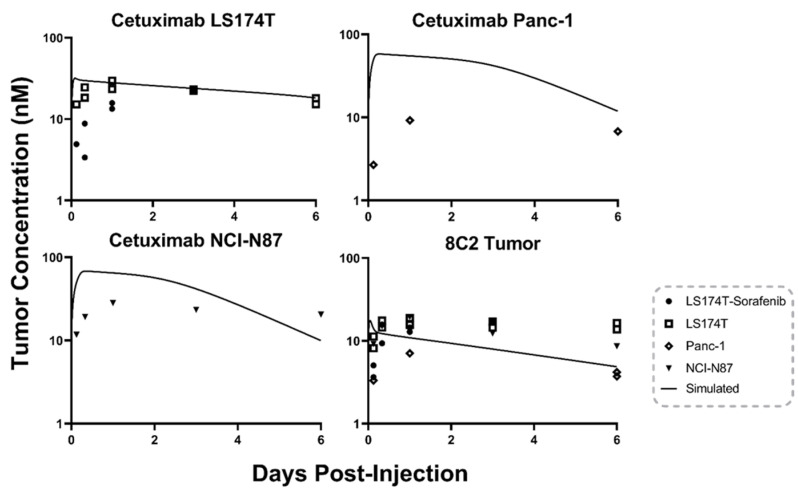
Base PBPK Model Prediction: The tumor concentration over time profile predictions made using the base PBPK model for 8C2 (bottom right) and cetuximab. Points represent the observed antibody concentration for a single xenograft. Cetuximab predictions are separated into three panels as cell-line specific EGFR concentrations were incorporated into the cetuximab model predictions.

To emulate clinical heterogeneity, four tumor models were utilized to increase the variability of 8C2 and cetuximab tumor pharmacokinetics. The first model is the colorectal cancer cell line LS174T, which has been extensively used for the development of our murine PBPK models [7,26]. Previous investigations by our group demonstrated that administration of 50 mg/kg sorafenib every other day to mice bearing LS174T tumors decreased the mean tumor vascular density and decreased tumor uptake of an anti-CEA antibody [27]. The third tumor model included in this study employed Panc-1, a pancreatic adenocarcinoma cell line. Panc-1 xenografts develop a relatively dense intra-tumoral matrix, and direct correlations between Panc-1 xenograft collagen content and the DCE-MRI parameter characterizing extracellular volume (Ve) have been reported [20]. Due to slower than expected tumor growth, only seven mice bearing Panc-1 tumors were included in this study. The fourth xenograft model considered in this work employed the gastric carcinoma cell line NCI-N87, which was co-administered with matrigel. Matrigel has been previously reported to impact nanotherapeutic uptake and penetration in comparison to a standard isotropic tumor model [28]. The tumor to plasma ratios between the four tumor models ranged from 0.08–0.26 for 8C2 treated tumors and from 0.12–0.48 for cetuximab treated tumors (Table 1). Antibody tumor concentrations for individual xenografts and base PBPK model predictions are provided in Figure 3. Base PBPK simulations for 8C2, as shown in the bottom right panel of Figure 3, captured the observed mean tumor data with a trend of overpredicting the xenografts with a low tumor/plasma ratio at early timepoints (3 and 8 h) and underpredicting tumor concentrations for xenografts with high tumor/plasma ratios at terminal timepoints (3 and 6 days). Base PBPK model predictions for cetuximab tumor pharmacokinetics are separated by tumor model as cell-line specific values of EGFR expression were incorporated into the model predictions. LS174T cetuximab tumor concentrations are well predicted by the base model with an MPE of 27.27%. The low error for the LS174T group may be explained by the fact the PBPK model was qualified using the same tumor animal model [7]. LS174T tumors treated with 50 mg/kg Sorafenib Q2D had decreased tumor concentrations of cetuximab at the 3 and 8 h timepoints in comparison to the non-treated LS174T tumors leading to model overprediction and an MPE of 291.20%. Cetuximab tumor concentrations for the NCI-N87 xenografts were over predicted at early timepoints (1, 3, and 24 h) with an MPE of 167.76%. Panc-1 cetuximab tumor concentrations were overpredicted across the three data points collected with an MPE of 769.68%.

### 2.2. DCE-MRI Fitting

**Figure 4:**
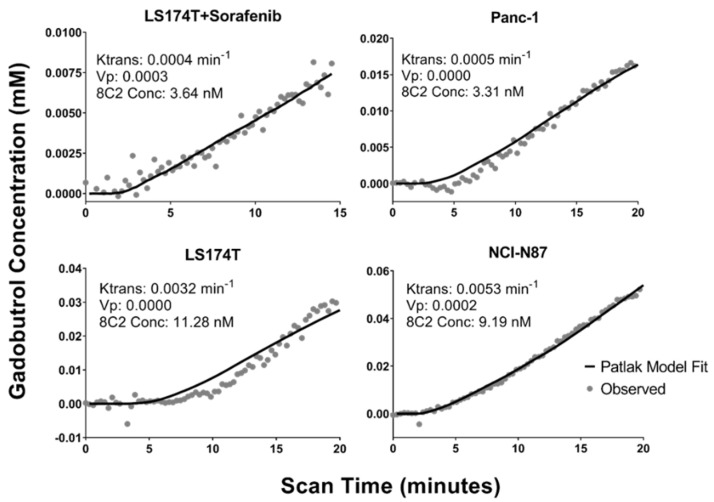
Representative DCE-MRI Data: Observed gadobutrol tumor concentrations (gray dots) and Patlak model fittings (solid black lines) are shown for xenograft tumors that were collected three hours after 8C2 administration. Fit Patlak model parameters for K^trans^ and Vp are provided for each tumor in addition to the observed 8C2 concentration at 3 h post-administration.

MR images were obtained in individual mice, with significant heterogeneity observed in the tumor contrast enhancement between xenografts. Heterogeneity in the AIF was also observed between mice, which is likely the result of difficulties at the injection site; however, this variability was controlled with the use of an individual AIF for each mouse as described in the methods. Figure 4 provides the observed tumor concentrations of gadobutrol in a representative xenograft bearing mouse for each of the four tumor models and the corresponding Patlak model fitting. The inset for each plot contains the individual best-fit values for the DCE-MRI parameters K^trans^ and Vp and the observed tumor concentration of 8C2 in the same xenograft at a 3 h terminal timepoint. In the example data provided, the LS174T + sorafenib tumor and the Panc-1 tumor have a fit K^trans^ value that is ~10-fold lower than the LS174T tumor and the NCI-N87 tumor and an observed 8C2 tumor concentration that is ~3-fold lower.

### 2.3. MRI-PBPK Covariate Modeling

**Figure 5:**
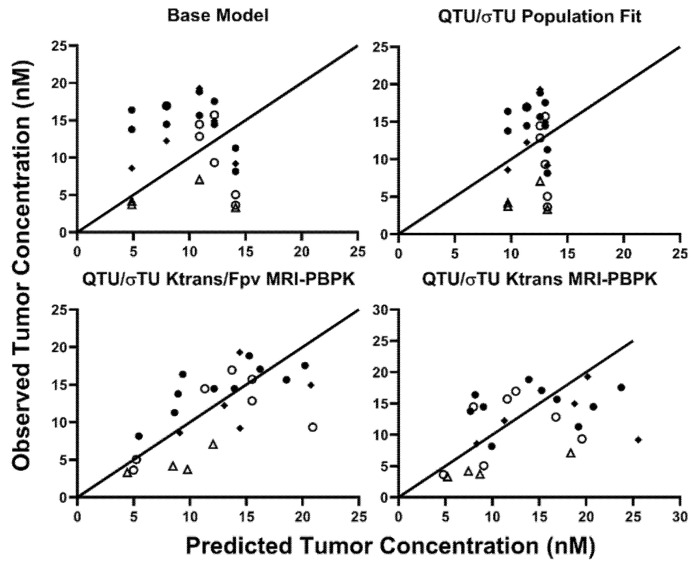
Observed vs. Predicted 8C2 Tumor Concentrations: Predicted vs. observed plots demonstrating the unity of predictions with the base model, population fit model, and the two-best covariate-integrated models. Tumor data in LS174T tumors following treatment with Sorafenib are represented in open circles, naïve LS174T tumors are depicted in closed circles. Panc-1 tumors are represented by an upright open triangle, and NCI-N87 data are represented by closed diamonds.

The PBPK tumor compartment considers many of the passive transport processes within solid tumors; however, only the PBPK parameters that relate to the same physiology as the DCE-MRI parameters were evaluated for MRI-PBPK covariate relationships. K^trans^ is the volume transfer coefficient and is sensitive to tumor blood flow, vascular surface area, and vasculature permeability. K^trans^ was evaluated as a covariate on PBPK parameters representing the tumor blood flow (QTU) and the tumor vasculature reflection coefficient (σ_TU_^V^). The DCE-MRI parameter Vp is the fractional plasma volume. The Vp was very small or 0 for many tumors in this study, which may be the result of poor tumor vasculature development or contrast agent injection difficulties. As a result, Vp was not able to be evaluated as a covariate on the PBPK parameter VVtu without model fitting error. Therefore, Vp was also considered as a covariate alone or paired with K^trans^ on the PBPK parameters QTU and σ_TU_^V^. The three best objective fit criteria for 8C2 tumor data were obtained with the population fit for QTU and σ_TU_^V^ (AIC = 171.22) or with consideration of K^trans^ (AIC = 170.21) or K^trans^ and Vp (AIC = 160.22) as covariates on QTU and σ_TU_^V^. An MPE for 8C2 tumor pharmacokinetics of 64.76% was obtained with the base PBPK model. Fitting QTU and σ_TU_^V^ to the 8C2 tumor data without consideration of the MRI covariates decreased the MPE to 61.67%. The addition of K^trans^ as a covariate on both QTU and σ_TU_^V^ decreased the MPE to 51.67%, and the addition of K^trans^ and Vp on QTU/σ_TU_^V^ decreased the MPE to 36.26%. Observed vs. predicted 8C2 tumor concentrations for the base model, the population fit values for QTU/σ_TU_^V^ and the K^trans^ or K^trans^/Vp covariate models are shown in Figure 5.

**Figure 6:**
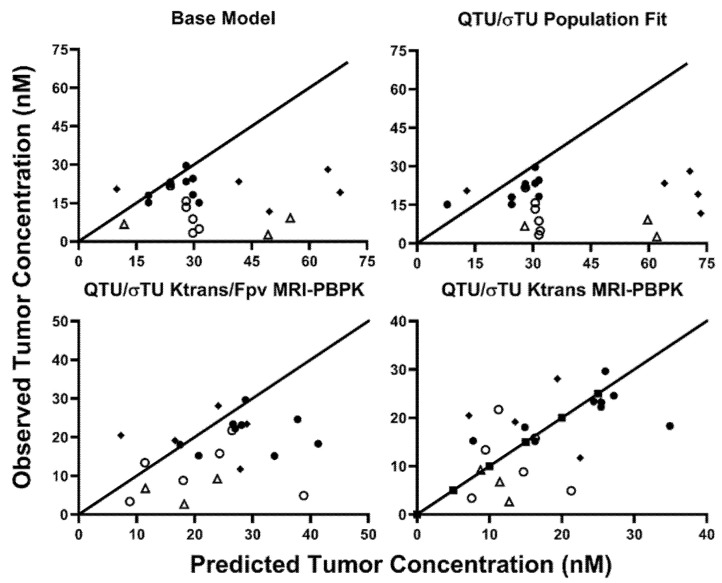
Observed vs. Predicted Cetuximab Tumor Concentrations: Predicted vs. observed plots demonstrating the unity of predictions with the base model, population fit model, and the two-best covariate-integrated models. Tumor data in LS174T tumors following treatment with Sorafenib are represented in open circles, naïve LS174T tumors are depicted in closed circles. Panc-1 tumors are represented by an upright open triangle, and NCI-N87 data are represented by closed diamonds.

The MRI-PBPK covariate relationships, established with 8C2, were implemented into the cetuximab PBPK model. Predictions were made on an individual xenograft basis using the base model, the population fit model, or the two covariate-integrated models using the individual observed values of K^trans^ and Vp in each xenograft. Observed vs. predicted tumor concentrations for each of the four PBPK predictions are provided in Figure 6. The MPE for cetuximab tumor pharmacokinetics using the base PBPK model was 223.71%. Updating the PBPK model with the 8C2 population fit values for QTU/σ_TU_^V^ increased the MPE to 283.30%. K^trans^ and Vp as covariates on QTU/σ_TU_^V^ decreased the MPE to 109.16%, whereas K^trans^ as a covariate on QTU/σ_TU_^V^ decreased the MPE to 65.02%. The disconnect between the best covariate relationship between 8C2 and cetuximab (K^trans^ alone vs. K^trans^ and Vp) is likely the result of the small group size of ~25 mice/antibody and experimental variability. Regardless, these results strongly support the hypothesis that DCE-MRI parameters can be utilized to improve patient-specific PBPK model predictions of therapeutic antibody tumor disposition.

## 3. Discussion

In this work, we evaluated the utility of quantitative MRI parameters to improve PBPK model predictions of cetuximab tumor pharmacokinetics in xenograft-bearing mice. To establish the initial relationship between MRI parameters and antibody tumor pharmacokinetics, the non-tumor binding anti-topotecan antibody 8C2 was used to avoid the impact of antigen-binding on tumor disposition. Target-mediated disposition is a major determinant of antibody uptake in solid tumors [29] and would impact the utility of the MRI-PBPK parameter relationship as MRI parameters are only sensitive to passive transport processes. Precise, patient-specific values of tumor cell antigen expression are often not available prior to mAb dosing. However, patient-specific, semi-quantitative immunohistochemical scores may be available in some settings. Of note, prior applications of our PBPK models have shown good predictions of mAb pharmacokinetics and pharmacodynamics with the estimation of receptor concentrations in tissues based on immunohistochemical (IHC) scores [4]. Using a population-averaged EGFR expression that was calculated using IHC scores [4] led to a similar MPE for the base PBPK model (252.07%) and the K^trans^ -QTU/σ_TU_^V^ covariate model (72.11%). The conversion of IHC scores to specific antigen expression values may be used in tandem with DCE-MRI for improved prediction of antibody pharmacokinetics for individual tumors in individual patients.

The ability to measure antibody tumor penetration is considered necessary to achieve personalized medicine for antibody-based therapies [30]. The use of DCE-MRI for a priori prediction of mAb pharmacokinetics seeks to address, in part, the missing clinical information needed to inform patient-specific antibody dosing protocols. Patient-specific PBPK predictions would allow individualized dosing regimens for a therapeutic mAb to obtain a desired tumor pharmacokinetic profile. Personalized predictions would be expected to provide a clear benefit in improving the outcomes of approved antibodies by optimizing current dosing protocols to a patient’s unique tumor physiology. A secondary benefit of personalized predictions is the potential application to clinical trial design. Therapies directed against solid tumors in Phase III trials have a likelihood of success of only 35.5% [31], with a lack of efficacy being the primary reason for failure [32]. Personalized predictions of antibody pharmacokinetics would allow stratification of patients based on individual tumor physiology prior to initiation of treatment. The benefit of considering patient-specific factors in clinical trial enrollment is exemplified by the anti-human epidermal growth factor receptor 2 (HER2) mAb trastuzumab. Retrospective subgroup analysis of the early clinical trials results for trastuzumab indicated that only patients with HER2 overexpression, defined as 3+ staining on IHC or >2x gene amplification on fluorescence in situ hybridization (FISH), responded to trastuzumab [33]. As only 20–30% of breast carcinomas overexpress the HER2 antigen [34], HER2 testing is currently required prior to patient enrollment for trastuzumab therapy, and several companion diagnostic assays for HER2 testing have been approved by the Food and Drug Administration (FDA) [33]. It is well appreciated that antigen density is a common factor influencing antibody efficacy, with biopsy and IHC staining a standard of care for many therapeutic antibodies. An additional example of patient-specific consideration is Kirsten rat sarcoma viral oncogene homolog (KRAS) profiling to select patients for cetuximab therapy. Multiple clinical trials with cetuximab in metastatic colorectal cancer observed a significant association between KRAS mutant status and cetuximab efficacy [35,36]. In these trials, ~40% of patients who had KRAS mutations were found to respond poorly to cetuximab [36]. As of 2009, the FDA requires KRAS exon 2 wildtype screening as a pre-requisite for cetuximab and panitumumab therapy. DCE-MRI scanning with a concurrent PBPK model may allow further improvements in patient selection for therapeutic antibodies by accounting for factors such as vascular permeability and interstitial fluid pressure that cannot be evaluated as part of a post-biopsy assay.

Many pre-clinical studies have demonstrated that enhanced tumor uptake of mAb correlates with increased anti-tumor effect [37,38]. For most monoclonal antibodies, the dose used in the clinic is well below the maximum tolerated dose; therefore, enhancing antibody tumor uptake and penetration can be achieved through the administration of higher antibody doses. A recent retrospective analysis reported dosing protocols chosen for clinical testing are poorly defined and are not often justified with an efficacy-based rationale [39]. Our approach could supplant current dosing protocols by recommending antibody doses that would be predicted to obtain the desired tumor concentration v. time profile. A potential dosing guideline based on the PBPK approach would be to determine the antibody dose required to saturate all available antigen sites within an individual tumor, leading to the best anti-tumor effect possible. A theoretical modeling approach by Thurber et al. demonstrated that current dosing protocols for mAbs likely lead to antigen saturation for well-vascularized tumors; however, for poorly vascularized tumors with high antigen expression, current doses are <20% of that needed to saturate tumor antigen [40].

The MRI-PBPK approach may also be of value in predicting the impact of methods to enhance mAb tumor uptake and penetration. Many strategies to improve mAb tumor disposition have been developed pre-clinically [21,41,42,43,44,45]; however, evaluating the impact of a given approach on mAb tumor PK requires complicated experimental methods (i.e., radioactive tracers, LC-MS/MS) with tumor samples collected across a range of timepoints. The DCE-MRI-PBPK approach may help to predict the impact of a given enhancement strategy on mAb tumor disposition.

Several barriers to entry exist for the proposed strategy before clinical implementation. The first is gathering the necessary data for establishing a suitable covariate relationship in patients. Recently, Lu et al. evaluated the tumor uptake of fluorescently labeled panitumumab in human patients with head and neck carcinoma following DCE-MR imaging and tumor resection [46]. A significant inverse correlation between the tumor size that was delineated using the MR images and tumor uptake of panitumumab was observed [46]. Additionally, patients with high heterogeneity in tumor contrast agent uptake were also observed to have heterogenous distribution of panitumumab following tumor resection [46]. The results observed by Lu et al. support the hypothesis that DCE-MR imaging can be used to predict tumor uptake of therapeutic mAb and demonstrate that the data required to develop the PBPK-MRI relationship can be feasibly obtained in human clinical trials. Perhaps the ideal tumor target for the DCE-MRI PBPK approach is breast cancer. Breast cancer has many clinically approved mAb-based therapies, including trastuzumab, pertuzumab, margetuximab-cmkb, ado-trastuzumab emtansine, fam-trastuzumab deruxtecan-nxki, and sacituzumab govitecan. Serendipitously, DCE-MRI is often used in patients with breast cancer to evaluate tumor development and facilitate treatment planning [47,48,49]. Comparable to the method used by Lu et al., patients eligible for tumor resection through surgery that also undergo DCE-MRI can be administered mAb prior to surgery. Following resection mAb tumor concentrations can be determined using a fluorescent tracer [46,50,51] or using liquid chromatography tandem mass spectrometry [52]. For tumors that are unable to be removed surgically, antibody uptake may be evaluated using immune-positron emission tomography, which has been applied in several clinical trials to evaluate mAb tumor uptake [53,54,55,56]. An additional barrier to the clinical implementation of this approach is the standardization of the DCE-MRI analysis protocol. Although DCE-MRI is commonly used clinically, standardization of quantitative metrics in the clinic has proven difficult. The choice of contrast agent, image analysis approach, MRI imaging sequence, and kinetic model can all lead to site-to-site variability in DCE-MRI parameters. The quantitative imaging biomarker alliance (QIBA) has provided recommendations on DCE-MRI analysis for clinical studies with the aim of reducing the site-to-site variability and may be considered a base protocol for any future clinical application of the DCE-MRI:PBPK approach.

Here, the utility of quantitative-MRI-based parameters to improve the prediction of cetuximab pharmacokinetics in xenograft tumor models was evaluated. The incorporation of the DCE-MRI parameter K^trans^ as a covariate on the PBPK parameters QTU/σ_TU_^V^ decreased the PBPK MPE for cetuximab tumor pharmacokinetics from 223.71 to 65.02%. As DCE-MRI is a common non-invasive technique, this approach holds promise for clinical translation to personalize antibody dosing regimens and improve patient selection for approved antibodies and antibodies being evaluated in clinical trials. Additional studies are warranted to evaluate if the DCE-MRI approach is applicable across tumor types, mAb therapies, and additional macromolecular drug delivery formats.

## 4. Materials and Methods

### 4.1. Antibody

Hybridoma cells expressing 8C2, a murine anti-topotecan IgG1, were cultured in a 1 L spinner flask containing serum-free media (Life Technologies, Carlsbad, CA, USA). 8C2 mAb was purified from cell media using a HiTrap Protein G column (Life Technologies, Carlsbad, CA, USA, on an NGC Quest 10 chromatography system (Bio-Rad, Hercules, CA, USA). Cetuximab was purchased from Millard Fillmore Hospital Pharmacy (Amherst, NY, USA).

### 4.2. Anti-Angiogenesis Agent

Sorafenib tosylate was purchased from Santa Cruz Biotechnology (Dallas, TX, USA). The drug was dissolved in dimethyl sulfoxide (DMSO), and aliquots were stored at −20 °C.

### 4.3. Xenograft Cell Lines

The following EGFR positive human cancer cell lines were purchased from American Type Culture Collection (ATCC, Manassas, VA, USA): LS174T (CL-188), a colorectal adenocarcinoma cell line, and Panc-1 (CRL-1469), a pancreatic epithelioid carcinoma. The gastric carcinoma cell line NCI-N87 (CRL-5822) was generously provided by Dr. Dhaval Shah’s laboratory. The cells were cultured following cell-line-specific ATCC recommendations.

### 4.4. Animals

Immune-deficient (NU/J) male mice, 4–6 weeks of age, were purchased from the Jackson Laboratory (Indianapolis, IN, USA). Mice were placed on sterile potassium iodide (KI) water (0.2 g/L) two days prior to injection of the radio-iodinated antibody to block thyroid uptake of free iodine. All mice were handled according to University at Buffalo’s Institutional Animal Care and Use Committee protocol.

### 4.5. Establishment of Xenografts

NU/J mice were injected subcutaneously into the right flank with either: LS174T cells suspended in sterile phosphate-buffered saline pH 7.4 (PBS) (2.0 × 10^6^ cells/mouse), Panc-1 cells suspended in base media (2.0 × 10^6^ cells/mouse), or NCI-N87 cells suspended in a 1:1 matrigel (Thermo Fisher Scientific, Waltham, MA, USA, CB-40234):base medium solution (5 × 10^6^ cells/mouse). Sorafenib-treated LS174T-bearing mice were administered IP injections of sorafenib tosylate every two days, starting four days post-implantation, at a dose of 50 mg/kg.

### 4.6. MR Imaging

A total of 49 xenograft-bearing mice underwent MRI at the University at Buffalo Center for Biomedical Imaging using a 20 cm diameter horizontal-bore 9.4-Tesla magnet (Biospec 94/20 USR, Bruker Biospin, Billerica, MA, USA) equipped with a gradient coil supporting 440 mT/m gradient strength and 3440 T/m/s maximum linear slew rate (BGA-12S HP; Bruker Biospin). The scanner was operated with ParaVision (version 5.1; Bruker Biospin). Prior to the image acquisition, mice were anesthetized with 3–4% isoflurane in 1 L/min of 100% medical-grade oxygen and immobilized on the MRI bed using gauze tape to reduce motion. Mouse respiration rate, heart rate, arterial oxygen saturation, and body temperature were monitored continuously with an SPO_2_-sensor attached to the animal’s tail, a respiration pillow, and a rectal temperature probe (model 1025, SA Instruments, Stony Brook, NY, USA). The isoflurane concentration and the temperature of an integrated warm water bath in the animal bed (Thermo Fisher Scientific, Waltham, MA, USA) were regulated to maintain mouse respiration rate and core body temperature. MRI sequences were obtained using the approach described by Gaustad et al. for assessing the effect of bevacizumab on xenograft tumors in mice [23]. Anatomical imaging was performed with a 2D T2-TurboRARE sequence using the following parameters: TE = 35 ms; TR = 2500 ms; 2 averages; RARE factor 8; 8 slices of 0.7 mm thickness; 128 × 128 matrix with 0.234 × 0.234 mm^2^ resolution; 100 kHz bandwidth; 1.4 kHz Gaussian fat suppression. Pre-contrast T1 values were determined using a 2D saturation recovery sequence (RAREVTR) with the following parameters: TE = 8.5 ms; TR = 200, 400, 800, 1500, 3000, and 5000 ms; RARE Factor 2; 66 kHz bandwidth; 8 slices with 0.7 mm thickness; and 0.234 × 0.234 mm^2^ in-plane resolution (128 × 128 matrix). A set of 70 DCE 3D gradient echo images were obtained with a time resolution of 17 s per image. The parameters used for the DCE-MRI sequence are: TE = 2 ms; TR = 10 ms; 20 degrees flip; 128 × 128 × 14 matrix with voxel size of 0.234 × 0.234 × 1 mm^3^; 64% partial Fourier in read direction; 104 kHz bandwidth; 1.4 kHz Gaussian fat suppression. A 0.06 M solution of Gadobutrol was administered at a dose of 5 μL/gram bodyweight two minutes after the initialization of the DCE scan sequence through the tail vein catheter.

### 4.7. Plasma and Tumor Pharmacokinetics

One day following MRI, 26 mice were administered 8C2 (10 LS174T, 7 LS174T+Sorafenib, 5 NCI-N87, and 4 Panc-1), and 23 mice were administered cetuximab (9 LS174T, 6 LS174T + Sorafenib, 5 NCI-N87, and 3 Panc-1). Cetuximab and 8C2 were injected into the retro-orbital plexus at a dose of 1 mg/kg with a 400 μCi/kg tracer dose of ^125^I-mAb. Cetuximab and 8C2 were radiolabeled with ^125^I through a modified chloramine-T method [57]. Mice were sacrificed for blood and tumor collection at 3 h, 8 h, 1 day, 3 days, and 6 days. Blood samples were centrifuged for 5 min at 500× *g*, plasma was collected, and TCA precipitated as described in our prior work [58]. Antibody concentrations in plasma and tumor tissue were determined through gamma counting with corrections for background radioactivity and radioactive decay.

### 4.8. Pharmacokinetic Modeling of DCE-MRI Images

Quantitative DCE-MRI analyses are often performed with the Extended-Tofts model [18,59,60]; however, accurate fitting of the reflux rate parameter Kep is dependent on the time of MRI acquisition and the tumor kinetics of the contrast agent. As no appreciable loss of contrast agent was observed during the time course of image acquisition, the Patlak model [61] was used for this analysis. The Patlak model is similar to the Extended-Tofts model with the assumption that no reflux of contrast agent from the interstitial space to the vascular space occurs [30]; therefore, Kep is not a fit parameter in the Patlak model. Individual tumor modeling with the Patlak pharmacokinetic model was performed in three steps. First, pre-contrast tumor relaxivity values (T_10_) were determined on a whole tumor basis using the parametric modeling module of ROCKETSHIP [62] using a tumor region of interest defined in MRIcron (Chris Rorden, University of South Carolina, Columbia, SC, USA). Second, an arterial plasma input function was defined on an individual mouse basis through manual selection and averaging of signal intensity versus time profiles of 4 voxels that showed rapid contrast enhancement at the time of gadobutrol injection using FIJI [63]. Dynamic contrast-enhanced scans were opened in 3D-Slicer [64], and tumor regions were defined using the segmentation editor. Signal intensity-over-time profiles of the tumor region were obtained using the multivolume explorer package. Exported signal intensity for the plasma and tumor space was converted to contrast agent concentration using Supplementary Equations (S1)–(S5). The Patlak pharmacokinetic model (Supplementary Equation (S6)) was fit to the contrast agent time profiles observed for individual mice with the corresponding AIF for each mouse using the Excel solver function through the minimization of the squared error.

### 4.9. Base Model PBPK Predictions

**Figure 7:**
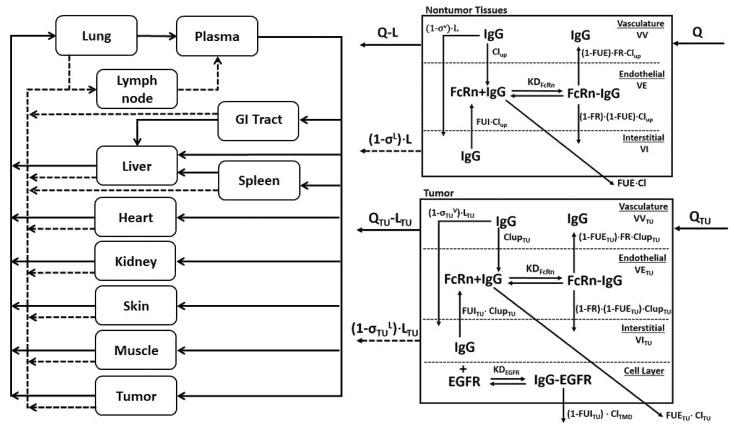
Schematic representation of the PBPK Model: The PBPK model is shown as a schematic diagram with solid lines representing plasma flow to and from tissues and dashed gray lines representing lymph flow. B. Each tissue compartment is composed of three sub-compartments representing vascular, endothelial, and interstitial spaces. Q and L represent tissue plasma and lymph flow, and σ^V^ and σ^L^ represent the vasculature and lymph reflection coefficients. Endosomal uptake is represented as Clup, and endosomal recycling is the product of Clup and the fraction of FcRn bound mAb that is returned to the vasculature, abbreviated as FR. KD_FcRn_ is the equilibrium dissociation constant for FcRn-mAb binding. Organ-specific elimination of unbound mAb in the endothelial space (FUE) is denoted as Cl. C. The tumor sub-compartment structure is shown. A 4th layer representing the cellular fraction is added, allowing the antibody to bind its target antigen (i.e., EGFR) with the observed equilibrium dissociation constant (KD_EGFR_) with bound antigen eliminated through receptor internalization and degradation (Cl_TMD_).

A physiologically based pharmacokinetic model (Figure 7) previously described [7] was adapted to predict tumor concentration-over-time profiles for 8C2 and cetuximab. Parameters for the tumor space are provided in Table 2. Equations representing the tumor compartment of the PBPK model are provided in the supplementary information (Equations (S7)–(S12)). Tumor concentrations for 8C2 were simulated using previously identified parameter values that are provided in Table 2. Predictions for cetuximab tumor pharmacokinetics were made separately for the three xenograft cell line groups using values reported in the literature for EGFR expression in the individual cell lines (Table 2). Simulations completed with the base model were used to determine the mean prediction error for 8C2 and cetuximab tumor pharmacokinetics.

### 4.10. MRI-PBPK Covariate Modeling

The DCE-MRI parameters K^trans^ and Vp, which were obtained from fitting MR images for individual mice to the Patlak model, were evaluated as potential descriptor relationships on the PBPK parameters: σ_TU_^V^ and QTU. Descriptor relationships for QTU were evaluated using Equation (1), where θ represents the slope of the relationship between the MRI parameter and the PBPK parameter:

(1) QTU=θ×MRI Parameter

To prevent the vasculature reflection coefficient exceeding a value of 1, the σ_TU_^V^ relationship was fit using Equation (2):

(2) $$\sigma_{TU}^{v}=\frac{1}{1+\theta \times \mathrm{MRI}\;\mathrm{Parameter}}$$

MRI-PBPK relationships were established for the untargeted antibody, 8C2, using the MLEM function in Adapt 5 [71] with 60 expectation-maximization steps and 3000 iterations/step. The PBPK parameters σ_TU_^V^ and QTU were also fit to the observed 8C2 data (referred to hereafter as the population fit model), without consideration of the MRI parameters, to ensure model improvement from the MRI-PBPK relationship was not the result of parameter misspecification. Objective fit criteria, observed vs. predicted plots and mean prediction error was used to select the two best MRI-PBPK covariate relationships. Subsequently, the two covariate-integrated models were used to predict cetuximab tumor pharmacokinetics in individual xenograft bearing mice based on the observed MRI parameter values for each xenograft (i.e., 1 xenograft mouse = 1 set of MRI values = 1 unique MRI-PBPK prediction per covariate model). PBPK predictions that implemented the MRI-PBPK relationships were compared to predictions made with the base PBPK model and the population fit model using observed vs. predicted plots and the mean prediction error.

## Figures and Tables

**Table 1:** Antibody Tumor-to-Plasma Ratio by Tumor Model.

Antibody	Tumor Model	Tumor/Plasma Ratio	Cetuximab/8C2
8C2	LS174T	0.26	1.58
Cetuximab	LS174T	0.41	-
8C2	LS174T/Sorafenib	0.20	1.35
Cetuximab	LS174T/Sorafenib	0.27	-
8C2	NCI-N87	0.19	2.53
Cetuximab	NCI-N87	0.48	-
8C2	Panc-1	0.08	1.50
Cetuximab	Panc-1	0.12	-

**Table 2:** PBPK Model Tumor Parameter Values and Definitions.

Parameter	Value	Units	Definition
Q_TU_	1 × 10^−4^	L/min	Tumor blood flow [7]
L_TU_	4 × 10^−6^	L/min	Tumor lymph flow [7]
Clup_TU_	8.18 × 10^−9^	L/min	Initial tumor uptake clearance [14]
σ_TU_^V^	0.734	-	Tumor vascular reflection coefficient [7]
σ_TU_^L^	0.2	-	Lymph reflection coefficient [7]
Cl_TU_	8.96 × 10^−9^	L/min	Initial clearance from endothelial space [14]
KD_FcRn_	7.5 × 10^−7^	M^–1^	FcRn-mAb KD [7]
C_FcRn_	1.64 × 10^−5^	M	Tumor FcRn concentration [14]
FR	0.715	-	Fraction of FcRn bound antibody recycled [7]
kgrowth	8.08 × 10^−5^	min^–1^	Tumor growth rate [7]
VI_TU_	1.38 × 10^−4^	L	Initial Interstitial Volume [14]
VE_TU_	1.25 × 10^−6^	L	Initial Endothelial Volume [14]
VV_TU_	1.75 × 10^−5^	L	Initial Vasculature Volume [14]
KD_EGFR_	1.5 × 10^−10^	M^–1^	Cetuximab-EGFR KD [4]
Kint	1.38 × 10^−3^	min^–1^	EGFR Internalization Rate [65,66]
Cl_TMD_	Kint × VI_TU_	L/min	Cetuximab Bound EGFR Clearance
C_EGFR_ (NCI-N87)	1.14 × 10^−7^	M	EGFR Tumor Concentration [67,68,69]
C_EGFR_ (Panc-1)	9.24 × 10^−8^	M	EGFR Tumor Concentration [70]
C_EGFR_ (LS174T)	3.53 × 10^−8^	M	EGFR Tumor Concentration [67,68]

## Data Availability

The data presented in this study are available on request from the corresponding author.

## Supplementary Materials

The following are available online at https://www.mdpi.com/article/10.3390/ijms23020679/s1.
