# Supplementary material for: Dynamic Contrast-Enhanced Magnetic Resonance Imaging for the Prediction of Monoclonal Antibody Tumor Disposition
Source: Int J Mol Sci. 2022 Jan 8;23(2):679. doi: 10.3390/ijms23020679 (PMC8775965; doi:10.3390/ijms23020679)
Supplement: Supplementary file 1 [file ijms-23-00679-s001.zip › ijms-1501765-supplementary.pdf]

International Journal of *Molecular Sciences*

MDPI

Supplementary Materials

Dynamic Contrast-Enhanced Magnetic Resonance Imaging for the Prediction of Monoclonal Antibody Tumor Disposition

Brandon M. Bordeau [1], Joseph Ryan Polli [1], Ferdinand Schweser [2,3], Hans Peter Grimm [4], Wolfgang F. Richter [4] and Joseph P. Balthasar [1,*]

1 Department of Pharmaceutical Sciences, University at Buffalo, 450 Pharmacy Building, Buffalo, NY 14214, USA; bmbordea@buffalo.edu (B.M.B.); jrpolli@buffalo.edu (J.R.P.)
2 Buffalo Neuroimaging Analysis Center, Department of Neurology, School of Medicine and Biomedical Sciences, University at Buffalo, Buffalo, NY 14203, USA; schweser@buffalo.edu
3 Clinical and Translational Science Institute. Center for Biomedical Imaging, University at Buffalo, Buffalo, NY 14203, USA
4 Roche Pharmaceutical Research and Early Development, Pharmaceutical Sciences, Roche Innovation Center Basel, F. Hoffmann-La Roche Ltd., Grenzacherstrasse 124, 4070 Basel, Switzerland; hans_peter.grimm@roche.com (H.P.G.); wolfgang.richter@roche.com (W.F.R.)
* Correspondence: jb@buffalo.edu; Tel: +1-716-645-4807

Equations Used to Convert T1 Signal to Gadobutrol Concentration

$$\boldsymbol{E10 = e^{-TR/T10}} \tag{S1}$$

$$\boldsymbol{B = \frac{(1 - E10)}{1 - Cos(FA) \times E10}} \tag{S2}$$

$$\boldsymbol{A = B\frac{SI(t)}{SI(0)}} \tag{S3}$$

$$\boldsymbol{\frac{1}{T1(t)} = -\frac{1}{TR} ln(\frac{1 - A}{1 - cos(FA) \times A})} \tag{S4}$$

$$\boldsymbol{C(t) = \frac{\frac{1}{T1(t)} - \frac{1}{T10}}{RGd}} \tag{S5}$$

Patlak Pharmacokinetic Model

$$\boldsymbol{C(t) = Vp \times Cp(t) + Ktrans \int_0^t Cp(\tau)\, d\tau} \tag{S6}$$

TR = Repetition time, T10 = Relaxivity of mouse blood at 9.4 Tesla [1], FA = flip angle, SI = Signal intensity, RGd = Relaxivity of Gadobutrol, T1(t) = observed relaxivity of tissue at time t, Cp(t) = plasma concentration of gadobutrol at time = t, Vp = Fractional Plasma volume, and Ktrans = volume transfer constant.

Tumor Compartment Equations

Tumor Vascular Space

$$\frac{dCV_{TU}}{dt} = Q_{TU} \times \frac{\mathrm{C_{PL}}}{VV_{TU}} + FR \times (1 - \mathrm{FUE}_{TU}) \times \mathrm{CLup_{TU}} \times \frac{\mathrm{CTE}_{TU}}{VV_{TU}} - CLup_{TU} \times \frac{\mathrm{CV}_{TU}}{VV_{TU}} - (1 - \sigma^{V}_{TU}) \times \mathrm{L}_{TU} \times \frac{\mathrm{CV}_{TU}}{\mathrm{VV}_{TU}} - (Q_{TU} - L_{TU}) \times \frac{\mathrm{CV}_{TU}}{\mathrm{VV}_{TU}};\ IC = 0 \tag{S7}$$

Tumor Endothelial Space

$$\frac{dCTE_{TU}}{dt} = CLup_{TU} \times \frac{\mathrm{CV}_{TU}}{\mathrm{VE}_{TU}} - FUE_{TU} \times \mathrm{CL}_{TU} \times \frac{\mathrm{CTE}_{TU}}{\mathrm{VE}_{TU}} - (1 - FUE_{TU}) \times \mathrm{CLup_{TU}} \times \frac{\mathrm{CTE}_{TU}}{\mathrm{VE}_{TU}} + \mathrm{CLup_{TU}} \times \mathrm{FUI_{TU}} \times \frac{\mathrm{CI_{TU}}}{\mathrm{VE}_{TU}};\ IC = 0 \quad \text{(S8)}$$

Tumor Interstitial Space

$$\frac{dCI_{TU}}{dt} = (1 - \sigma^{V}_{TU}) \times \mathrm{L}_{TU} \times \frac{\mathrm{CV}_{TU}}{\mathrm{VI}_{TU}} - (1 - \sigma^{L}_{TU}) \times \mathrm{L}_{TU} \times \mathrm{FUI}_{TU} \times \frac{\mathrm{CI}_{TU}}{\mathrm{VI}_{TU}} + (1 - FR) \times (1 - \mathrm{FUE}_{TU}) \times \mathrm{CLup_{TU}} \times \frac{\mathrm{CTE}_{TU}}{\mathrm{VI}_{TU}} - \mathrm{FUI}_{TU} \times \mathrm{CLup}_{TU} \times \frac{\mathrm{CI}_{TU}}{\mathrm{VI}_{TU}} - (1 - FUI_{TU}) \times \mathrm{Cl_{TMDD}} \times \frac{\mathrm{CI_{TU}}}{VITU};\ IC = 0 \quad \text{(S9)}$$

IgG Free Fraction Endothelial Space (FcRn binding)

$$FUE_{TU} = 1 - \frac{\left((KD_{FcRn} + C_{FcRn} + CTE_{TU}) - \sqrt{(KD_{TU} + C_{FcRn} + CTE_{TU})^2 - 4 \times \mathrm{CTE}_{TU} \times C_{FcRn}}\right)}{2 \times \mathrm{CTE_{TU}}} \quad \text{(S10)}$$

IgG Free Fraction Interstitial Space (Cetuximab EGFR binding)

$$FUI_{TU} = 1 - \frac{\left((KD_{EGFR} + C_{EGFR} + CI_{TU}) - \sqrt{(KD_{EGFR} + C_{EGFR} + CI_{TU})^2 - 4 \times \mathrm{CI}_{TU} \times C_{EGFR}}\right)}{2 \times \mathrm{CI_{TU}}} \quad \text{(S11)}$$

Tumor Growth Dependent Parameter (VV_{TU}, VE_{TU}, VI_{TU}, CLup, $CLup_{TU}$)

$$P(t) = P(0) \times e^{kgrowth \times \mathrm{T}} \quad \text{(S12)}$$

REFERENCES

1. Walker-Samuel, S.; Roberts, T.A.; Ramasawmy, R.; Burrell, J.S.; Johnson, S.P.; Siow, B.M.; Richardson, S.; Goncalves, M.R.; Pendse, D.; Robinson, S.P.; et al. Investigating Low-Velocity Fluid Flow in Tumors with Convection-MRI. Cancer Res. 2018, 78, 1859–1872, https://doi:10.1158/0008-5472.CAN-17-1546.
